# Oral immunization of a non-recombinant *Lactococcus lactis* surface displaying influenza hemagglutinin 1 (HA1) induces mucosal immunity in mice

**DOI:** 10.1371/journal.pone.0187718

**Published:** 2017-11-06

**Authors:** Pui-Fong Jee, Vunjia Tiong, Meng-Hooi Shu, Jing-Jing Khoo, Won Fen Wong, Raha Abdul Rahim, Sazaly AbuBakar, Li-Yen Chang

**Affiliations:** 1 Department of Medical Microbiology, Faculty of Medicine, University of Malaya, Kuala Lumpur, Malaysia; 2 Tropical Infectious Diseases Research and Education Centre (TIDREC), University of Malaya, Kuala Lumpur, Malaysia; 3 Department of Cell and Molecular Biology, Faculty of Biotechnology and Biomolecular Sciences, Universiti Putra Malaysia, Serdang, Selangor, Malaysia; New York Blood Center, UNITED STATES

## Abstract

Mucosal immunization of influenza vaccine is potentially an effective approach for the prevention and control of influenza. The objective of the present study was to evaluate the ability of oral immunization with a non-recombinant *Lactococcus lactis* displaying HA1/L/AcmA recombinant protein, LL-HA1/L/AcmA, to induce mucosal immune responses and to accord protection against influenza virus infection in mice. The LL-HA1/L/AcmA was orally administered into mice and the immune response was evaluated. Mice immunized with LL-HA1/L/AcmA developed detectable specific sIgA in faecal extract, small intestine wash, BAL fluid and nasal fluid. The results obtained demonstrated that oral immunization of mice with LL-HA1/L/AcmA elicited mucosal immunity in both the gastrointestinal tract and the respiratory tract. The protective efficacy of LL-HA1/L/AcmA in immunized mice against a lethal dose challenge with influenza virus was also assessed. Upon challenge, the non-immunized group of mice showed high susceptibility to influenza virus infection. In contrast, 7/8 of mice orally immunized with LL-HA1/L/AcmA survived. In conclusion, oral administration of LL-HA1/L/AcmA in mice induced mucosal immunity and most importantly, provided protection against lethal influenza virus challenge. These results highlight the potential application of *L*. *lactis* as a platform for delivery of influenza virus vaccine.

## Introduction

Influenza virus infection results in respiratory illnesses and contributes to a high rate of morbidity and mortality in humans, particularly children, the elderly and immunocompromised individuals. Three influenza pandemics had occurred in the 20^th^ century: 1918 Spanish flu, 1957–1958 Asian influenza and 1968 Hong Kong influenza, while one recently occurred in the 21^st^ century: 2009 pandemic H1N1 [1]. Besides pandemic influenza, seasonal epidemics of influenza is also of great concern, as it causes approximately 3–5 million of severe illness and 250,000–500,000 deaths annually worldwide [2]. Controlling the spread of influenza remains a major challenge. It is undeniable that vaccines, which confer protection against influenza, are most effective.

Currently, there are three types of influenza vaccine available in the market: an inactivated influenza virus vaccine (IIV), a live attenuated influenza virus vaccine (LAIV) and a recombinant vaccine. The IIV is a good inducer of systemic immune response but it is ineffective at stimulating mucosal immune response [3]. LAIV on the other hand, induces both systemic and mucosal immune responses [4]. However, there is an associated risk of the attenuated virus reverting back to its highly virulent form and thus causing an infection [3]. A case of LAIV transmission to a recipient was previously reported [4]. In addition, a study had shown that LAIV increased risk of wheezing within 42 days after vaccination in children <12-months-old [5]. Recombinant vaccine is potentially of advantage especially in the event of a pandemic due to its quick production potential [6]. This vaccine is administered intramuscularly and therefore, induces immune response similar to IIV [7]. However, a few serious adverse events associated with this vaccine such as vasovagal syncope, pericardial effusion and Bell’s palsy were previously reported [8]. Taken together, influenza vaccines that are currently available present several drawbacks, including their limited ability at stimulating mucosal immunity without compromising safety issues. As influenza virus initiates infection at the respiratory tract mucosal surface, it would be advantageous to have a vaccine that is able to induce mucosal immune response, mainly the secretory IgA (sIgA).

One of the current strategies in mucosal vaccine research targeting stimulation of mucosal immunity is oral administration of an antigen using *Lactococcus lactis* as a delivery platform. *L*. *lactis* is a generally recognized as safe bacterium. It has been applied in mucosal vaccine research due to its non-colonizing and non-pathogenic properties. More recently, *L*. *lactis* is being extensively explored as effective oral-based vaccine vehicles to deliver antigens of several viruses [9–15], bacteria [16–20] and parasites [21]. Studies have shown that *L*. *lactis* expressing and displaying antigens were capable of inducing strong systemic as well as mucosal immune responses [9–12, 21–24].

Considering the role of mucosal immunity in protection against influenza, we have previously developed a non-recombinant *L*. *lactis* surface displaying influenza A (H1N1) 2009 hemagglutinin (HA1) using N-acetylmuramidase (AcmA) as a binding domain to anchor HA1 on the bacterial cell wall [25]. The amount of HA1 bound on *L*. *lactis* was improved using a single-chain variable fragment peptide linker (Gly_4_Ser)_3,_ to link HA1 to the AcmA binding domain. In the present study, we extended our previous work by optimizing the binding parameters, the amount of HA1 recombinant protein added to *L*. *lactis*, the duration of binding and the buffer used for binding. We then assessed the specific immune response elicited upon oral immunization of the non-recombinant *L*. *lactis* surface displaying HA1 (LL-HA1/L/AcmA) in mice. In addition, the protective efficacy of LL-HA1/L/AcmA against a lethal dose challenge with influenza virus was also assessed.

## Materials and methods

### Ethics statement

All procedures involving animals were reviewed and approved by the Faculty of Medicine-Institutional Animal Care and Use Committee (FOM-IACUC) with ethics reference no. 2014-01-07/MMB/R/JPF. Ketamine-xylazine (ketamine: 80–120 mg/kg; xylazine: 10–16 mg/kg) was used to anesthetize the animals in this study. An overdose of ketamine-xylazine (ketamine: 240–360 mg/kg; xylazine: 30–48 mg/kg) was given to euthanize the animals in this study.

### Optimization of *L*. *lactis* binding conditions

*L*. *lactis* displaying HA1/L/AcmA recombinant protein was prepared as previously described [25]. Briefly, an overnight culture of *L*. *lactis* MG1363 was subcultured in GM17 broth and incubated at 30°C until the OD_600_ reading reached 0.5. The *L*. *lactis* culture (2 ml) was centrifuged at 2,000 ×g for 10 min and the cell pellet obtained was resuspended in fresh GM17 broth (0.6 ml). Subsequently, refolded HA1/L/AcmA recombinant protein (100 μl) was prepared as previously described [25] and added to the cell suspension and incubated at 30°C for 2 h. The cells were then washed with phosphate buffered saline (PBS) thrice by centrifugation at 2,000 ×g for 10 min. The cell pellet was eventually resuspended in 200 μl of PBS. Optimizations were performed to improve the binding of HA1/L/AcmA to *L*. *lactis* by varying the binding parameters, specifically the amount of recombinant protein (25, 20, 15, 10 and 5 μg) added to equal number of *L*. *lactis* cells, the duration of binding (1, 2, 3 and 4 h), and the type of binding buffer (GM17 and PBS). Other factors that may influence the binding of recombinant protein onto *L*. *lactis*, including pH and temperature were not examined as these factors may affect the integrity of the cell membrane and other biological processes of *L*. *lactis*, which may then affect the binding of recombinant proteins on the bacterial cell wall.

After binding, the *L*. *lactis* cells pre-mixed with the HA1/L/AcmA were stained and analyzed using flow cytometry as previously described [25]. Briefly, cells were fixed with 200 μl of 4% (w/v) paraformaldehyde for 20 min and blocked with 200 μl of 3% (w/v) bovine serum albumin (BSA) for 30 min. Subsequently, the cells were incubated with 200 μl of mouse monoclonal anti-polyhistidine (Sigma-Aldrich, USA) at 1:1,000 dilution for 1 h, followed by incubation with 200 μl of Alexa Fluor® 488 goat anti-mouse IgG (Invitrogen Life Technologies, USA) at 1:500 dilution in the dark for 1 h. The cells were washed with PBS between steps thrice and recovered by centrifugation at 2,000 ×g for 10 min. The cells were eventually suspended in 1 ml of PBS for examination of stained cells using a fluorescence-activated cell sorting (FACS) CANTO ™ II Flow cytometer (BD Biosciences, USA). A total of 5×10^4^ cells falling into the gate defined on the forward angle light scatter-side angle light scatter plot were acquired to determine the positive cell count and mean fluorescence intensity (MFI).

### Immunogen preparation

*L*. *lactis* displaying HA1/L/AcmA recombinant protein was prepared using the optimal conditions as determined earlier and scaled-up by proportionally increasing the amount of recombinant protein and *L*. *lactis* cells to be used in the binding. The *L*. *lactis* displaying HA1/L/AcmA was resuspended in endotoxin-free PBS to a concentration of 1×10^11^ CFU/ml and 5×10^11^ CFU/ml for study groups A and B, respectively. The constructed non-recombinant *L*. *lactis* displaying HA1/L/AcmA was named LL-HA1/L/AcmA.

### Mice immunizations

Six to eight-weeks-old specific-pathogen-free female BALB/c mice were purchased from InVivos Pte Ltd. (Singapore). Animals were housed in specific-pathogen-free conditions with free access to food and water in accordance with Association for the Assessment and Accreditation of Laboratory Animal Care International and FOM-IACUC guidelines of University of Malaya. A total of two study groups were performed to evaluate the immunogenicity of LL-HA1/L/AcmA. In study group A, mice (n = 8) were immunized orally with 0.1 ml of 1×10^10^ CFU of LL-HA1/L/AcmA (25 µg/dosage) or endotoxin-free PBS by intra-gastric lavage for 3 consecutive days. The immunization regimen was repeated thrice at 2 weeks intervals. In study group B, mice (n = 8) were immunized orally with 0.1 ml of 5×10^10^ CFU of LL-HA1/L/AcmA (125 μg/dosage), HA1/L/AcmA (125 μg/dosage) or endotoxin-free PBS for 3 consecutive days. The immunization regimen was repeated thrice at 2 weeks intervals. As a positive control, HA1/L/AcmA (50 μg/dosage) emulsified with complete Freund’s adjuvant (CFA; Sigma-Aldrich, USA), HA1/L/AcmA-FA, was administered subcutaneously to a group of mice (n = 8). The immunization was repeated thrice at 2 weeks intervals using incomplete Freund’s adjuvant (IFA; Sigma-Aldrich, USA) instead of CFA.

### Sample collection

In study group A, blood and faeces were collected 2 weeks after the last immunization. Thereafter, the mice were euthanized by intraperitoneal injection of an overdose of ketamine-xylazine and samples such as small intestine, bronchoalveolar lavage (BAL) and nasal fluid were collected. In study group B, only blood and faecal pellets were collected. The mice in study group B were not euthanized, but kept for viral challenge.

Briefly, blood sample was collected via tail vein and serum was obtained by centrifugation at 1,000 ×g for 5 min. Faecal pellets (100 mg) were resuspended in 0.5 ml sterile PBS containing 1 mM phenylmethanesulfonyl fluoride (PMSF), vortexed vigorously and faecal extract was obtained by centrifugation at 10,000 ×g for 5 min. Small intestine was harvested and flushed thrice with 1 ml PBS containing 1 mM PMSF. The sample from small intestine was then obtained following centrifugation at 10,000 ×g for 5 min. BAL [26, 27] and nasal fluids [26] were collected as previously described with minor modifications. Briefly, mouse trachea was cannulated with an intravenous catheter (BD Bioscience, USA) and connected to a syringe. BAL fluid was obtained by flushing the lungs with 1 ml cold PBS for 5 times. The washing step was repeated once and lavage fluid recovered from two washings was pooled. Nasal fluid was obtained by flushing the nasopharynx with 1 ml cold PBS for 5 times and nasal wash fluid was recovered from the mouse nostril. The BAL and nasal fluids were centrifuged at 200 ×g for 5 min, and the supernatant samples were collected. All samples were stored in -20°C for subsequent analysis.

### Enzyme-linked immunosorbent assay (ELISA) for detection of mucosal and systemic immune response

HA1-specific IgG and IgA antibodies were detected by enzyme-linked immunosorbent assay (ELISA) following the protocol described by Joan et al. [28] with some modifications. Briefly, 96-well microtiter plate (Costar Corporation, USA) was coated with 100 μl of HA1/L/AcmA recombinant protein (1 μg/well) at 4°C for overnight. The wells were blocked with 3% (w/v) BSA for 1 h. Sera or mucosal samples were added and incubated for 1 h. Bound antibody was detected using HRP-conjugated goat anti-mouse IgG (Abcam, UK) at 1:10,000 dilution or HRP-conjugated rat anti-mouse IgA (Abcam, UK) at 1:1,000 dilution. The plates were washed 3 times with PBS between steps. TMB peroxidase substrate system (KPL, USA) was added for detection. After incubation for 20 min, TMB stop solution (KPL, USA) was added and absorbance value at 450 nm was measured using a microplate reader (Tecan, Switzerland). The assay was performed with 3 technical replicates.

### H1N1 virus challenge experiment

A stock of mouse adapted A/TN/1-560/2009-MA2(H1N1) influenza virus was kindly provided by Dr. Richard J. Webby (St. Jude Children’s Research Hospital, USA). At 18 days following the last immunization, all mice in study group B were fully anesthetized by intraperitoneal injection of ketamine-xylazine and intranasally inoculated with 50 μl of 10 LD_50_ of A/TN/1-560/2009-MA2(H1N1) influenza virus. After inoculation, body weight and survival rate of the mice were monitored daily up to 14 days. Mice that showed body weight loss of >20% were considered to have reached the experimental end-point and were euthanized by intraperitoneal injection of an overdose of ketamine-xylazine. No adverse reactions or mortality occurred outside of the humane endpoint was observed throughout the study.

### Statistics

Statistical analysis and graphical representations were performed using GraphPad Prism 5 software (GraphPad Software, USA). Data analysis was performed using Student’s t test for the comparison of two groups. The log-rank (Mantel-Cox) test was used for statistical comparison of the survival between groups.

## Results

### Optimization of the binding of HA1/L/AcmA recombinant protein to *L*. *lactis*

**Fig 1:**
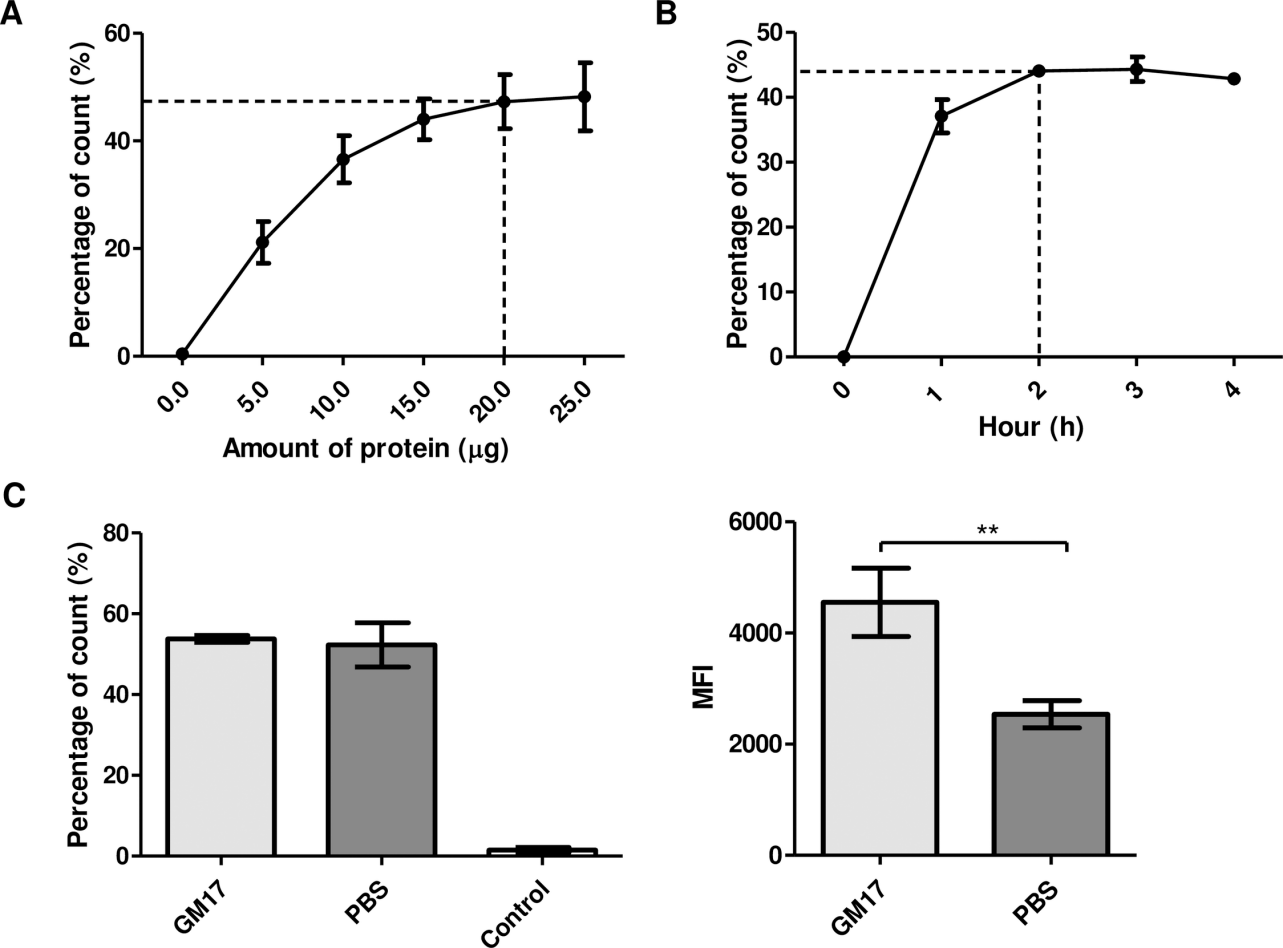
Binding optimization of HA1/L/AcmA recombinant protein to *L*. *lactis*. (A) The percentage of *L*. *lactis* surface displaying HA1/L/AcmA recombinant protein after incubation with different amounts of HA1/L/AcmA recombinant protein. (B) The percentage of *L*. *lactis* surface displaying HA1/L/AcmA recombinant protein after incubation with HA1/L/AcmA recombinant protein for 1 h, 2 h, 3 h, and 4 h, respectively. (C) The percentage and mean fluorescence intensity (MFI) value of *L*. *lactis* surface displaying HA1/L/AcmA recombinant protein after incubation with HA1/L/AcmA recombinant protein in GM17 and PBS, respectively. The data represents mean ± standard deviation. Asterisk indicates statistically significant differences between groups (**P<0.01).

*L*. *lactis* surface displaying HA1/L/AcmA recombinant protein was immunofluorescence-labeled with Alexa Fluor 488. The percentage of Alexa Fluor 488-positive gated cells (*L*. *lactis* surface displaying HA1/L/AcmA) increased steadily when increasing amount of HA1/L/AcmA recombinant protein was added to 1–3×10^9^ CFU of *L*. *lactis* (Fig 1A). The percentage of Alexa Fluor 488-positive gated cells reached plateau when 20 μg of HA1/L/AcmA recombinant protein was used. The results suggested that the optimum amount of HA1/L/AcmA recombinant protein to be incubated with 1–3×10^9^ CFU of *L*. *lactis* cells for surface display was 20 μg.

The percentage of *L*. *lactis* surface displaying HA1/L/AcmA recombinant protein following incubation of *L*. *lactis* with 20 μg of HA1/L/AcmA recombinant protein in GM17 for 1 h, 2 h, 3 h, and 4 h was examined. The percentage of Alexa Fluor 488-positive gated cells reached plateau when 20 μg of HA1/L/AcmA recombinant protein was incubated with *L*. *lactis* cells for 2 h, suggesting that incubation for 2 h was optimum for surface display of HA1/L/AcmA recombinant protein (Fig 1B). The binding of HA1/L/AcmA recombinant protein on *L*. *lactis* started to dissociate after an incubation period of 3 h.

The suitability of the buffer used for binding of HA1/L/AcmA recombinant protein to *L*. *lactis* was also evaluated. The percentage of *L*. *lactis* surface displaying HA1/L/AcmA recombinant protein after 2 h incubation with HA1/L/AcmA recombinant protein in GM17 and PBS was 53.8±0.9% and 52.3±5.5%, respectively, suggesting that the number of *L*. *lactis* cells detected to have HA1/L/AcmA on its cell wall was similar in both buffers (Fig 1C). Therefore, the MFI value of *L*. *lactis* surface displaying HA1/L/AcmA recombinant protein was examined. The MFI value obtained for HA1/L/AcmA recombinant protein binding in GM17 (4552±614.9) was significantly higher in comparison to PBS (2538±243.4). This suggested that more recombinant proteins were bound per *L*. *lactis* cells in GM17 and hence, was a better binding buffer.

### Immune response induced by oral immunization with LL-HA1/L/AcmA in mice

**Fig 2:**
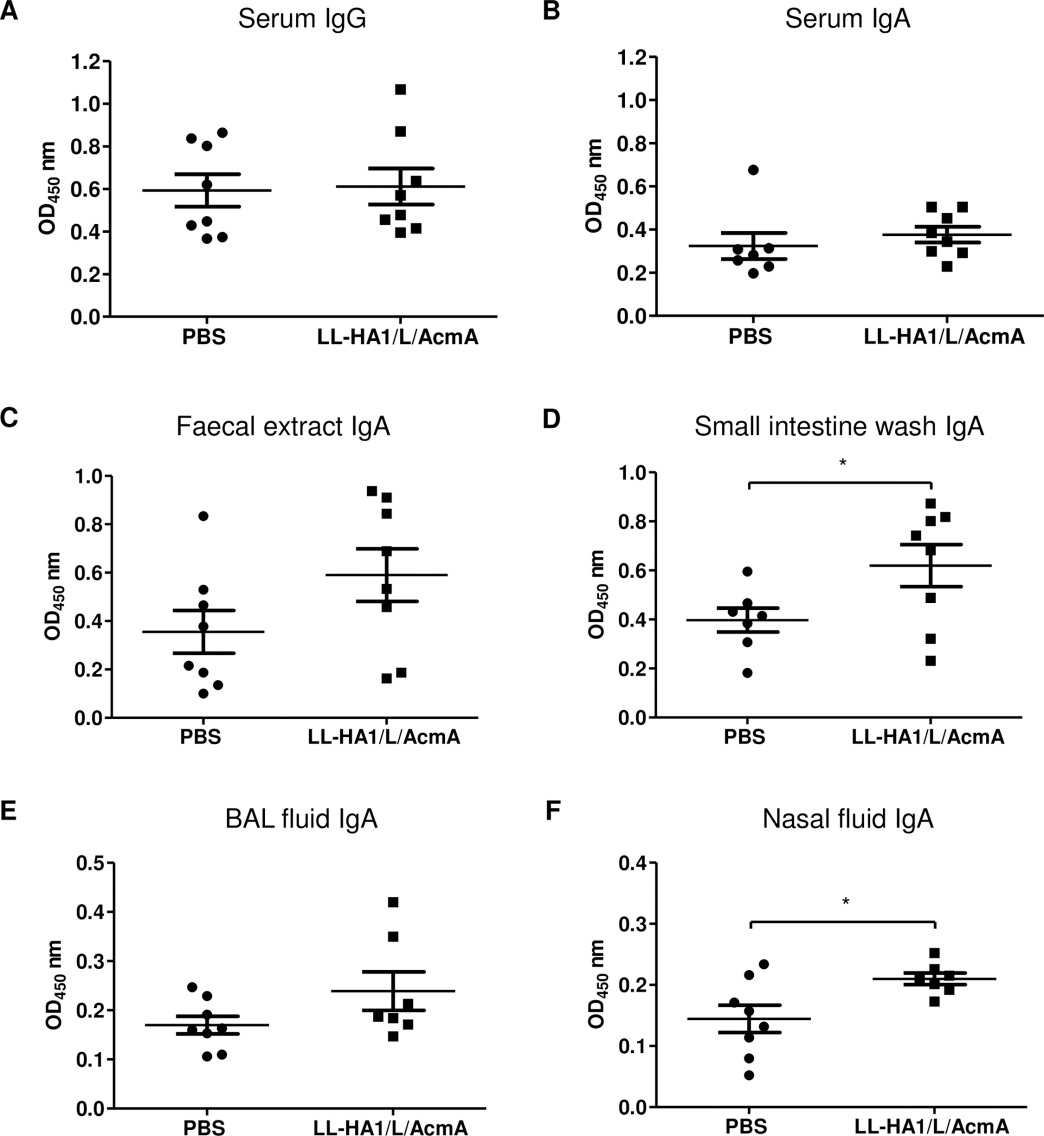
HA1/L/AcmA-specific IgG and IgA detected by ELISA. Mice (8 mice/group) were orally immunized with PBS or LL-HA1/L/AcmA, and samples were collected 2 weeks after the last immunization. HA1/L/AcmA-specific (A) IgG and (B) IgA in serum at 1:10 dilution were determined, in addition to HA1/L/AcmA-specific IgA in (C) faecal sample (1:5 dilution), (D) small intestine wash (neat), (E) BAL fluid (neat) and (F) nasal fluid (neat). One significant outlier was removed from the data set for serum IgA and small intestine wash of PBS-treated group, as well as BAL and nasal fluid of LL-HA1/L/AcmA-treated group (Grubbs’ test, p<0.05), respectively. Data are presented as mean ± standard error of the mean. Asterisks indicate statistically significant differences between the corresponding groups (*p<0.05).

Study group A was a preliminary study to evaluate HA1-specific immune responses towards *L*. *lactis* surface displaying HA1/L/AcmA recombinant protein, LL-HA1/L/AcmA. HA1/L/AcmA-specific serum IgG and IgA were measured as the main readout for systemic immunity. There were no differences in the serum IgG (Fig 2A) and IgA (Fig 2B) in mice immunized with LL-HA1/L/AcmA (mean OD: 0.61±0.08 and 0.38±0.04, respectively) compared to the PBS-treated group (mean OD: 0.59±0.07 and 0.32±0.06, respectively). One significant outlier was detected in the data set for serum IgA of PBS-treated group using Grubbs’ test (p<0.05) and was excluded in the statistical analysis. Overall, results suggested that there were no stimulation of serological immune responses upon oral immunization of LL-HA1/L/AcmA in mice, or the immune responses were below the detection limit of the assay.

HA1/L/AcmA-specific sIgA in faecal extract, small intestine wash, BAL fluid and nasal fluid were measured as main readout of mucosal immunity. The HA1/L/AcmA-specific sIgA in faecal sample (Fig 2C) was noted to be higher, but there was no statistically difference between mice immunized with LL-HA1/L/AcmA (mean OD: 0.59±0.11) and the PBS-treated group (mean OD: 0.36±0.09). In the data set for small intestine wash, one significant outlier was detected in PBS-treated group using Grubbs’ test (p<0.05) and was removed from the statistical analysis. The HA1/L/AcmA-specific sIgA in small intestine wash (Fig 2D) of mice immunized with LL-HA1/L/AcmA (mean OD: 0.62±0.09) was detected to be significantly higher (p<0.05) than PBS-treated group (mean OD: 0.40±0.05). In both data set for BAL and nasal fluid of mice immunized with LL-HA1/L/AcmA, one significant outlier was detected using Grubbs’ test (p<0.05) in each of these groups and were excluded in the statistical analysis. The HA1/L/AcmA-specific sIgA in BAL fluid (Fig 2E) was noted to be higher, but not statistically different in mice immunized with LL-HA1/L/AcmA (mean OD: 0.24±0.03) when compared to the PBS-treated group (mean OD: 0.17±0.01). In contrast, mice immunized with LL-HA1/L/AcmA developed a significantly higher HA1/L/AcmA-specific sIgA in nasal fluid (p<0.05) (mean OD: 0.21±0.01) compared to PBS-treated group (mean OD: 0.14±0.02) (Fig 2F). Collectively, results suggested that oral immunization with LL-HA1/L/AcmA in mice stimulated a significant level of mucosal immunity in the gastrointestinal tract and respiratory tract.

In study group B, mice were immunized orally with a five-fold higher dosage of LL-HA1/L/AcmA to determine if a higher dose would elicit a greater immune response. For comparison, additional control groups of mice were included: mice orally administered with HA1/L/AcmA recombinant protein only (125 μg/dosage) and mice subcutaneously immunized with HA1/L/AcmA (50 μg/dosage) emulsified with Freund’s adjuvant (HA1/L/AcmA-FA).

**Fig 3:**
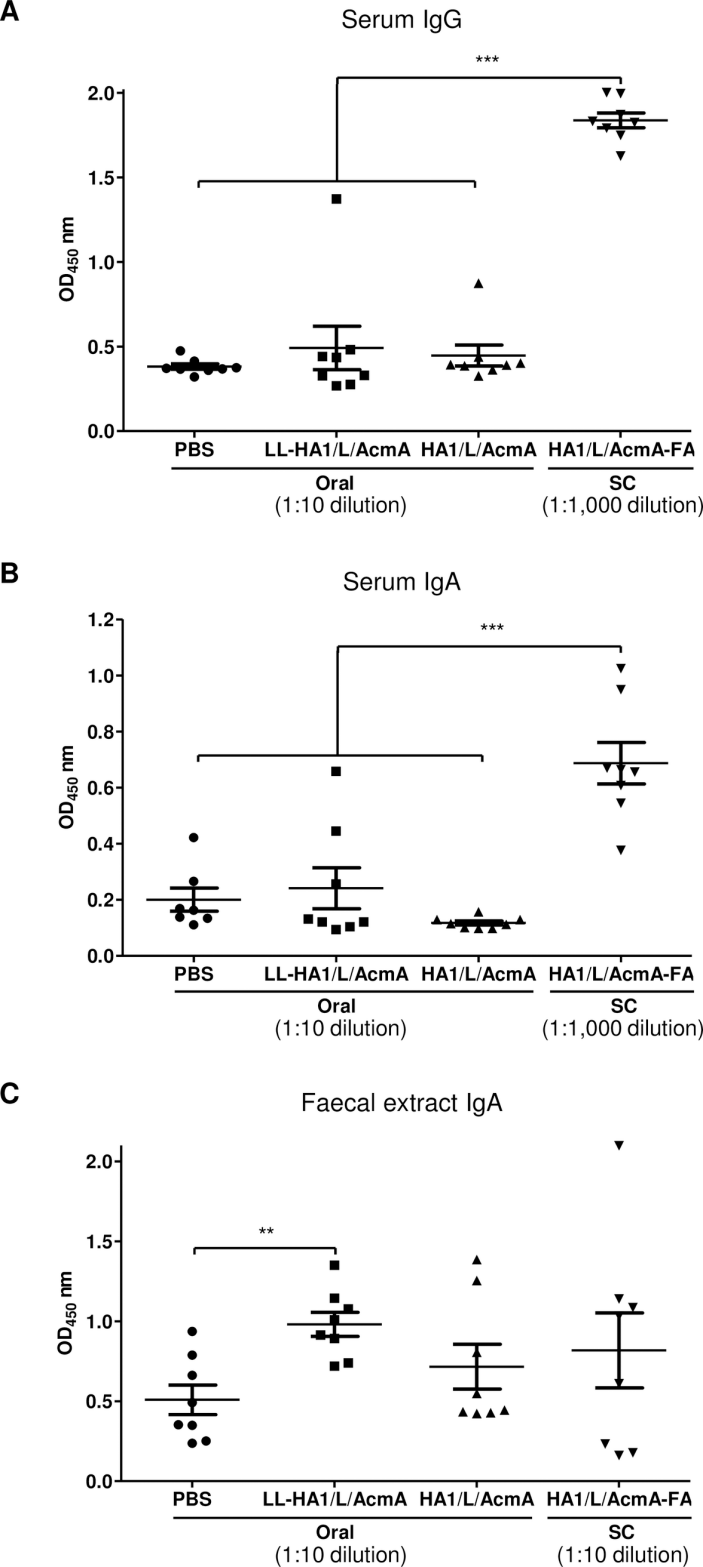
HA1/L/AcmA-specific serum IgG, serum IgA and faecal IgA detected by ELISA. Mice (8 mice/group) were orally immunized with PBS, LL-HA1/L/AcmA or HA1/L/AcmA, or subcutaneously (SC) immunized with HA1/L/AcmA-FA. Serum and faecal samples were collected 2 weeks after the last immunization. (A) HA1/L/AcmA-specific serum IgG, (B) serum IgA and (C) faecal IgA from individual mouse were determined. Data are presented as mean ± standard error of the mean. Asterisks indicate statistically significant differences between the corresponding groups (**p<0.01; ***p<0.001).

There were no significant differences in the HA1/L/AcmA-specific serum IgG (Fig 3A) and serum IgA (Fig 3B) for mice orally immunized with LL-HA1/L/AcmA (mean OD: 0.49±0.13 and 0.24±0.07, respectively) when compared to PBS-treated group (mean OD: 0.38±0.02 and 0.20±0.04, respectively). The same was observed for mice orally immunized with HA1/L/AcmA recombinant protein only (mean OD: 0.45±0.06 and 0.12±0.01, respectively) in comparison to PBS-treated group. One significant outlier was detected in the data set for serum IgA of PBS-treated group using Grubbs’ test (p<0.05) and was excluded in the statistical analysis. Another significant outlier was detected in the data set for serum IgG of mice immunized with LL-HA1/L/AcmA using Grubbs’ test (p<0.05), but was not removed as an outlier because this mouse showed detectable HA1/L/AcmA-specific serum IgG and IgA response, which increased with each subsequent immunization. There were also no significant differences in serum IgG and IgA levels between mice orally immunized with LL-HA1/L/AcmA and HA1/L/AcmA. In contrast, significantly highest serum IgG (Fig 3A) and IgA (Fig 3B) (p<0.001) were detected in the positive control group, which were mice immunized subcutaneously with HA1/L/AcmA-FA (mean OD: 1.84±0.04 and 0.69±0.07, respectively). Results suggested that there were no stimulation of serological immune responses upon oral immunization of LL-HA1/L/AcmA or HA1/L/AcmA in mice. Alternatively, the responses were below the detection limit of the assay. Nonetheless, subcutaneous immunization of HA1/L/AcmA-FA in mice stimulated good serological immune responses, suggesting that HA1/L/AcmA is highly antigenic.

Significantly higher HA1/L/AcmA-specific sIgA (Fig 3C) (p<0.01) was detected in faecal sample of mice orally immunized with LL-HA1/L/AcmA (mean OD: 0.98±0.07) than the PBS-treated group (mean OD: 0.51±0.09). A higher dosage of LL-HA1/L/AcmA administered in mice effectively improved mucosal immune response, and this is clearly indicated by the significant stimulation of sIgA detected in the faecal sample (Figs 2C and 3C). The HA1/L/AcmA-specific sIgA in faecal sample of mice orally immunized with LL-HA1/L/AcmA was also higher, but not statistically different compared to mice immunized with HA1/L/AcmA orally (mean OD: 0.72±0.14) or HA1/L/AcmA-FA subcutaneously (mean OD: 0.82±0.23) (Fig 3C). There was no significant difference in the detected HA1/L/AcmA-specific sIgA in faecal sample of mice orally immunized with HA1/L/AcmA from the PBS-treated group, suggesting HA1/L/AcmA without *L*. *lactis* as a carrier did not stimulate significant level of HA1/L/AcmA-specific sIgA. The same was noted for mice immunized subcutaneously with HA1/L/AcmA-FA as compared to PBS-treated group. Although mice immunized subcutaneously with HA1/L/AcmA-FA stimulated good serological immune responses, however, it did not stimulate significant level of sIgA in the faecal sample. Taken together, the results suggested that LL-HA1/L/AcmA is a potent inducer of mucosal immunity in the gastrointestinal tract upon oral immunization of LL-HA1/L/AcmA in mice, and *L*. *lactis* delivery platform is capable in stimulating mucosal immunity in the gastrointestinal tract.

### Protection against lethal challenge

**Fig 4:**
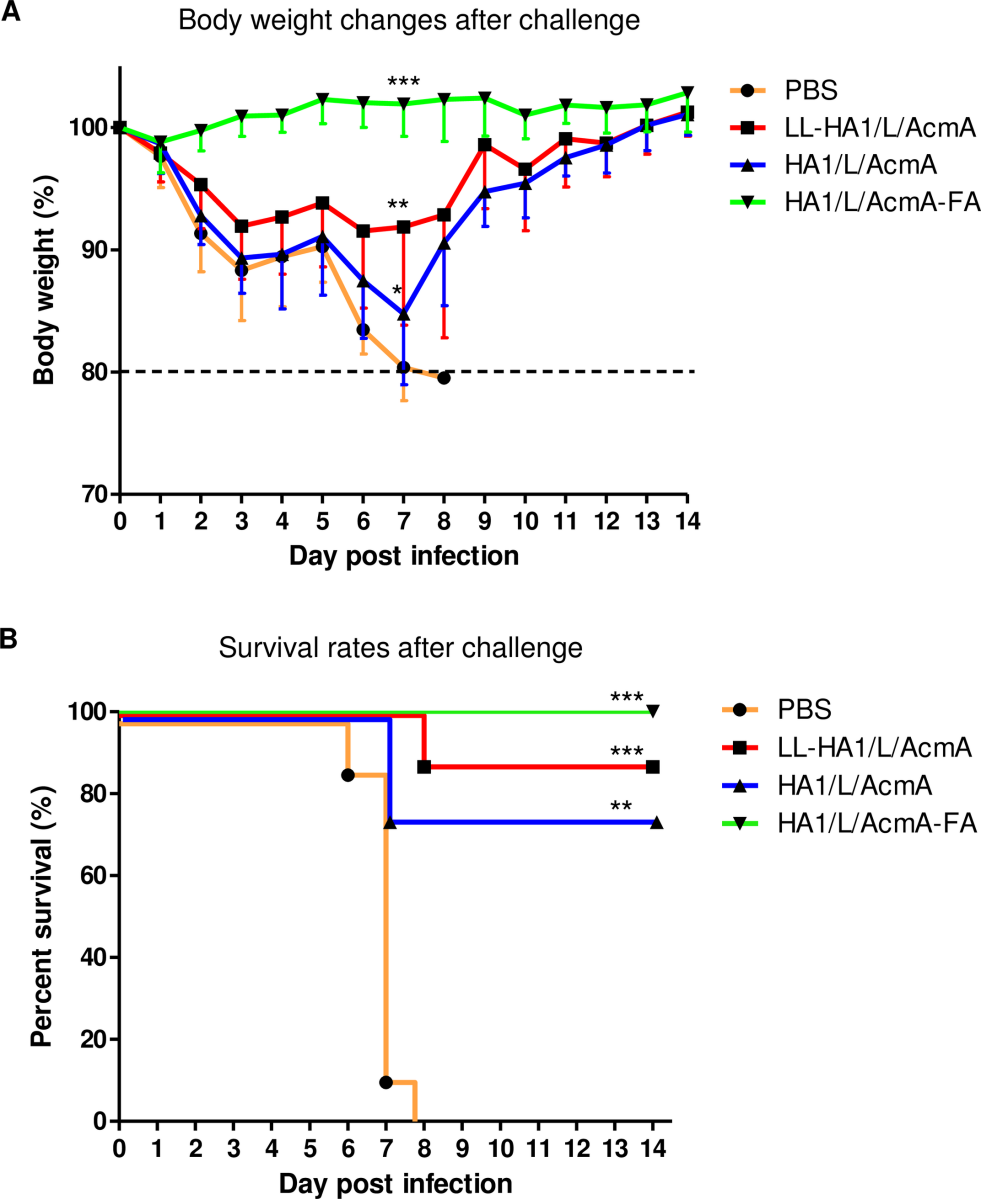
Body weight and survival rate of mice following lethal challenge with influenza virus. At 18 days following the last immunization, all mice in study group B were intranasally challenged with 10 LD_50_ of H1N1/A/TN/1-560/2009-MA2 virus. (A) Body weight and (B) survival of mice orally immunized with PBS, LL-HA1/L/AcmA or HA1/L/AcmA, or subcutaneously immunized with HA1/L/AcmA-FA, was monitored daily for 14 days following virus challenge. The body weight of mice on the day of viral challenge was used as the baseline weight to determine the body weight changes post-viral challenge. Asterisks indicate statistically significant differences in comparison with the PBS-treated group (*p<0.05; **p<0.01; ***p<0.001).

At 18 days following the last immunization, all mice in study group B were challenged intranasally with influenza virus to evaluate the protective efficacy of orally administered LL-HA1/L/AcmA against exposure to influenza virus. Changes in body weight and survival of mice were monitored daily as measure of disease severity. All non-immunized mice were susceptible to influenza infection, showing sickness such as decreased activity and huddling by day 3 post-infection (p.i.). Severe sickness such as hunched posture, ruffled fur and increased body weight loss of up to 20% were observed within day 8 p.i. (Fig 4A). The rate of body weight loss calculated by linear regression analysis was -2.7±0.1 and the maximum percentage change in body weight observed in individual mice over 14 days after lethal challenge with influenza virus was 20.6±0.1% (Table 1). In contrast, body weight of mice in LL-HA1/L/AcmA-treated and HA1/L/AcmA-treated group decreased until day 6 and 7 p.i., respectively, after which mice in both groups recorded an increase in their body weight till day 14 p.i. (Fig 4A). A lower rate of body weight loss was observed in the LL-HA1/L/AcmA-treated (-0.3±0.1) and HA1/L/AcmA-treated group (-0.6±0.1), respectively, as compared to non-immunized mice (-2.7±0.1) (Table 1). Significantly lower percentage change in body weight was noted in LL-HA1/L/AcmA-treated (11.6±2.5%) (p<0.01) and HA1/L/AcmA-treated group (15.7±1.8%) (p<0.05) than the non-immunized mice. In contrast, the positive control group, which were mice immunized subcutaneously with HA1/L/AcmA-FA did not show body weight loss upon exposure to influenza virus (Fig 4A). The mice instead recorded rate of body weight gain of 0.2±0.0 (Table 1). In addition, there was significantly lower percentage change in body weight (p<0.001) in this HA1/L/AcmA-FA-treated group (1.5±0.7%) compared to the non-immunized group (Table 1). These data indicated that mice orally immunized with LL-HA1/L/AcmA or HA1/L/AcmA resulted in significantly less morbidity upon exposure to influenza virus. Mice in the HA1/L/AcmA-treated group suffered more severe sickness as presented by highest rate of body weight loss and percentage change in body weight among the treated group. Subcutaneous immunization of HA1/L/AcmA-FA in mice did not result in any morbidity upon exposure to virus. Collectively, it was demonstrated that oral immunization of LL-HA1/L/AcmA is superior in reducing morbidity upon exposure to influenza virus compared to the HA1/L/AcmA without *L*. *lactis* as a carrier.

**Table 1:** Body weight and survival rate of immunized mice upon lethal challenge with influenza virus.

Immunogen	Rate of body weight loss^a)^	Rate of body weight recovery^b)^	Body weight loss, %^c)^	No. of survival (%)^d)^	Mean day of death, p.i.^e)^
PBS	-2.7±0.1	-	20.6±0.1	0/8 (0)	7.0±0.5
LL-HA1/L/AcmA	-0.3±0.1	0.3±0.1	11.6±2.5 **	7/8 (88) ***	8.0±0.0
HA1/L/AcmA	-0.6±0.1	0.3±0.1	15.7±1.8 *	6/8 (75) **	7.0±0.0
HA1/L/AcmA-FA	0.2±0.0	0.2±0.0	1.5±0.7 ***	8/8 (100) ***	>14.0

^a), b)^ Rate of body weight loss and body weight recovery was calculated by linear regression analysis. Data are presented as mean ± standard deviation.

^c)^ The maximum percentage change in body weight observed in individual mice over 14 days after lethal challenge with influenza virus. Data are presented as mean ± standard deviation.

^d)^ Total number of mice survived following lethal challenge with influenza virus. Percentage of mice survived following virus challenge is presented in parentheses.

^e)^ Day of death following lethal challenge with influenza virus. Data are presented as mean ± standard deviation.

Asterisks indicate statistically significant differences in comparison to the PBS-treated group (*p<0.05; **p<0.01; ***p<0.001).

The survival rate of immunized mice upon exposure to influenza virus was assessed. Significantly higher survival rate of mice was observed in LL-HA1/L/AcmA-treated (7/8, 88%) (p<0.001) and HA1/L/AcmA-treated group (6/8, 75%) (p<0.01) upon exposure to influenza virus than non-immunized group (Fig 4B; Table 1). Survival rate of mice immunized with HA1/L/AcmA-FA was the highest and significantly different (p<0.001) from non-immunized group, with all mice fully protected from the challenge (8/8, 100%). There were no differences in the survival between all the immunized groups. As for non-immunized group, all mice were not protected (0/8, 0%) and the mean day of death was 7.0±0.5 p.i. The mean day of death LL-HA1/L/AcmA-treated and HA1/L/AcmA-treated group was 8.0±0.0 p.i. and 7.0±0.0 p.i., respectively. Importantly, oral immunization of mice with LL-HA1/L/AcmA or HA1/L/AcmA, or subcutaneous immunization with HA1/L/AcmA-FA provided protection to the animals against a lethal challenge with influenza virus.

## Discussion

Influenza virus, a respiratory pathogen, contributes to a high rate of morbidity and mortality in humans globally. As such, while antiviral therapy has been available, vaccination is still the most effective strategy for the prevention and control of influenza. There is growing interest in mucosal vaccines due to their advantages over conventional injectable vaccines. Side effects associated with conventional vaccines, such as local reactions at the injection site are potentially reduced with implementation of mucosal vaccines. The need for needles during vaccine administration is also avoided, eliminating the possibility of blood transmissible infections [29, 30]. Moreover, mucosal vaccination can be easily administered without a trained personnel, thus considered a more favorable approach for mass vaccination [31].

Most pathogens, including influenza viruses, initiate infection by entering the human body through mucosal surfaces. Mucosal vaccination stimulates this natural infection and can provide local immune protection by stimulating sIgA at the mucosal surfaces. SIgA is the most abundant immunoglobulin isotype present in human secretions [32] and is effective in according protection against influenza virus infection [33]. It has been reported to be more important than IgG in the protection of upper respiratory tract, specifically the nose and trachea [34], primarily by reducing virus attachment and preventing internalization of the virus at the mucosal surfaces, thereby preventing infection [34, 35]. In addition, sIgA provides cross-protection against other subtypes of influenza virus [33, 36–38]. Cross-protection is particularly desirable due to the frequent antigenic changes that influenza viruses constantly undergo.

Of the various mucosal immunization approaches, *L*. *lactis* is being explored as an effective vaccine vehicle. We previously developed a recombinant *L*. *lactis* displaying influenza HA1 and evaluated the immunity elicited by this recombinant construct in mice [28]. This recombinant *L*. *lactis* carried the HA1 gene that was genetically introduced into a vector containing an antibiotic resistance gene. The presence of this antibiotic resistance gene could eventually be a matter of concern, particularly the transfer of its antibiotic resistance gene to another organism when *L*. *lactis* is released in the field [39]. An approach utilizing a non-recombinant *L*. *lactis* will be advantageous as it is likely to overcome this concern, and hence be better accepted by the public. We subsequently developed a non-recombinant *L*. *lactis* displaying influenza HA1, LL-HA1/L/AcmA [25], and evaluated its ability in the stimulation of mucosal immunity in this present study.

In the present study, findings of HA1/L/AcmA-specific sIgA in faecal extract, small intestine wash, BAL fluid and nasal fluid demonstrated that oral immunization with LL-HA1/L/AcmA in mice elicited significant level of mucosal immunity in the gastrointestinal tract and the respiratory tract. The result is consistent with previous studies showing that oral immunization with *L*. *lactis* displaying antigens induced sIgA at sites other than the gastrointestinal tract [22, 24, 40–42]. The results of sIgA at other mucosal sites could be due to intestinally derived IgA plasma cells expressing CCR10 homing receptor that migrated towards the CCL28 cytokine, which is secreted by mucosal epithelial tissues present at sites such as large intestine, stomach, trachea, bronchi, mammary glands and salivary glands [43]. These migrated intestinal IgA plasma cells then populate the mucosal sites, and secrete HA1/L/AcmA-specific sIgA. Therefore, oral immunization could lead to presence of antigen specific IgA in both intestinal and non-intestinal mucosal tissues [44, 45].

In study group B, higher dosage of LL-HA1/L/AcmA significantly increased the specific sIgA response compared to non-immunized group. The results suggested that sIgA response elicited by LL-HA1/L/AcmA could be improved in a dose-dependent manner. The observed specific sIgA response upon oral immunization with LL-HA1/L/AcmA was consistent with our previously reported findings in which specific sIgA was detected upon oral immunization with recombinant *L*. *lactis* expressing HA1 [28].

HA1/L/AcmA-specific serum IgG and IgA were almost absent following immunization with LL-HA1/L/AcmA, with the exception of one mouse (1/8) that elicited detectable serum IgG and IgA upon administration with a higher dosage of LL-HA1/L/AcmA. The result obtained was in contrast with several studies that had demonstrated oral immunization of antigen using *L*. *lactis* elicited specific IgG in serum in addition to specific sIgA in faecal extract [9, 11, 12, 46–48]. Although mice immunized orally with LL-HA1/L/AcmA elicited significant mucosal immune response only, and not systemic immune response, more importantly, it was demonstrated that LL-HA1/L/AcmA could provide up to 88% protection in mice against a lethal challenge with influenza virus. An earlier study on mice immunized orally with *L*. *lactis* expressing HA of influenza H5N1 showed significant increase in specific serum IgG and intestinal IgA, but IgA was not detected in tracheal mucosal and only 30% mice were protected upon viral challenge [46]. Therefore, in this study, the protection in mice was likely due to the presence of sIgA specific against HA1 in the respiratory tract, which was possibly activated upon oral immunization. It would be beneficial to evaluate the neutralization ability of the sIgA induced by LL-HA1/L/AcmA by measuring its capability to inhibit influenza virus A (H1N1) *in vitro*, however it could not be examined due to the limitation of samples obtained. Besides sIgA, there are other protective mechanisms, such as T cell-mediated immunity, which may play a protective role by clearing the established infection and reducing disease severity [49, 50]. Therefore, additional studies are needed to elucidate the specific mechanism of protection by LL-HA1/L/AcmA. Nonetheless, the findings obtained highlight the importance of mucosal immunity in the respiratory tract in according protection against influenza virus challenge.

In comparing the importance of *L*. *lactis* as an antigen carrier, mice immunized orally with LL-HA1/L/AcmA showed markedly improved immune response than HA1/L/AcmA recombinant protein only, without *L*. *lactis*. Mice immunized with LL-HA1/L/AcmA also suffered less sickness and body weight loss upon lethal challenge with influenza virus. These results were in agreement with several earlier studies that oral immunization with *L*. *lactis* displaying antigen is more efficient than a simple antigen alone oral immunization [15, 47, 51]. The potential adjuvant effects of using *L*. *lactis* in oral immunization could be attributed to the presence of bacterial components that can stimulate innate immunity, leading to adaptive immunity [52]. Besides, *L*. *lactis* presenting heterologous antigen as a particle to the immune system, is more superior to soluble antigen presented on its own, particularly if the vaccine is to be delivered orally [53]. As the present study is focused only on the protection of LL-HA1/L/AcmA in mice against homosubtypic influenza virus challenge, heterosubtypic influenza virus challenge can now be explored. Further investigation to surface display antigens from multiple influenza subtypes on a single *L*. *lactis* cell as candidate vaccine to render protection against a broad range of influenza subtypes can also be performed.

In conclusion, oral administration of *L*. *lactis* surface displaying HA1/L/AcmA recombinant protein induces specific sIgA response in mice that protected against homologous influenza virus challenge. These findings indicate the application of *L*. *lactis* as a platform for vaccine delivery and the importance of mucosal immunity in according protection against lethal challenge with influenza virus.
